# The Efficacy of Shugan Jianpi Zhixie Therapy for Diarrhea-Predominant Irritable Bowel Syndrome: A Meta-Analysis of Randomized, Double-Blind, Placebo-Controlled Trials

**DOI:** 10.1371/journal.pone.0122397

**Published:** 2015-04-08

**Authors:** Ya Xiao, Yanyan Liu, Shaohui Huang, Xiaomin Sun, Yang Tang, Jingru Cheng, Tian Wang, Fei Li, Yuxiang Kuang, Ren Luo, Xiaoshan Zhao

**Affiliations:** 1 Department of Traditional Chinese Medicine, Nanfang Hospital, Southern Medical University, Guangzhou, Guangdong, 510515, China; 2 School of Traditional Chinese Medicine, Southern Medical University, Guangzhou, Guangdong, 510515, China; 3 Department of Rheumatic diseases, The First Affiliated Hospital of Guangzhou University of Chinese Medicine, Guangzhou, 510405, China; 4 Digestive Department of Guangdong provincial hospital of TCM, Guangzhou, 510120, China; University Hospital Llandough, UNITED KINGDOM

## Abstract

**Background:**

Shugan Jianpi Zhixie therapy (SJZT) has been widely used to treat diarrhea-predominant irritable bowel syndrome (IBS-D), but the results are still controversial. A meta-analysis of randomized, double-blind, placebo-controlled trials was performed to assess the efficacy and tolerability of SJZT for IBS-D.

**Methods:**

The MEDLINE, EMBASE, Cochrane Library, the China National Knowledge Infrastructure database, the Chinese Biomedical Literature database and the Wanfang database were searched up to June 2014 with no language restrictions. Summary estimates, including 95% confidence intervals (CI), were calculated for global symptom improvement, abdominal pain improvement, and Symptom Severity Scale (BSS) score.

**Results:**

Seven trials (N=954) were included. The overall risk of bias assessment was low. SJZT showed significant improvement for global symptom compared to placebo (RR 1.61; 95% CI 1.24, 2.10; *P* =0.0004; therapeutic gain = 33.0%; number needed to treat (NNT) = 3.0). SJZT was significantly more likely to reduce overall BSS score (SMD –0.67; 95% CI –0.94, –0.40; *P* < 0.00001) and improve abdominal pain (RR 4.34; 95% CI 2.64, 7.14; *P* < 0.00001) than placebo. The adverse events of SJZT were no different from those of placebo.

**Conclusions:**

This meta-analysis suggests that SJZT is an effective and safe therapy option for patients with IBS-D. However, due to the high clinical heterogeneity and small sample size of the included trials, further standardized preparation, large-scale and rigorously designed trials are needed.

## Introduction

Irritable bowel syndrome (IBS) is associated with symptoms of chronic abdominal pain, bloating and disturbed defecation in the absence of any demonstrable biochemical and anatomical abnormality [1]. About 5%-22% of general population is affected by IBS among various countries [2,3]. IBS causes significant reductions in patients’ quality of life and daily activities and involves substantial healthcare costs [4–6].

Although great progress has been made in the understanding of irritable bowel syndrome, conventional treatment remains unsatisfied. A series of systematic reviews of published drug trials were performed by the American College of Gastroenterology Task Force [7]. The quality of evidence for certain antispasmodics was graded as poor, and for tricyclic antidepressants, selective serotonin reuptake inhibitors, non-absorbable antibiotics, and C-2 chloride channel activators as moderate [7]. The trials of 5HT3 antagonists and 5HT4 agonists were of good quality, but it was noteworthy that these drugs were associated with a potential risk of ischemic colitis and cardiovascular events respectively [7].

Due to chronicity and frequency of symptoms, many patients seek alternative treatments such as traditional Chinese medicine (TCM) [8–10]. TCM is characterized by syndrome differentiation. In this regard, “stagnation of liver energy and deficiency of spleen” is considered to be the basic pathogenic factor of diarrhea-predominant IBS (IBS-D) [11]. Relieving the suppressed liver and replenishing the spleen energy for anti-diarrhea (Chinese name in pinyin “Shugan Jianpi Zhixie”) is the most important therapy in the treatment of IBS-D [12]. *Tong Xie Yao Fang*, an ancient formula with function of Shugan Jianpi Zhixie, has been prescribed by TCM practitioners for a long time in the treatment of IBS-D [13]. There was a systematic review of *Tong Xie Yao Fang* for IBS reported in 2006 [14]. In the review, twelve studies were included, but none of them were double-blind, placebo-controlled trials. The authors concluded that no definitive conclusions could be drawn due to the poor quality of the primary trials. Recently, increasing numbers of well-designed trials assessing Shugan Jianpi Zhixie therapy (SJZT) for IBS have been published [12,15–21]. However, the current state of evidence of SJZT for IBS-D has so far been unknown. Therefore, we conducted a meta-analysis of randomized, double-blind, placebo-controlled trials to determine whether SJZT is beneficial to patients with IBS-D.

## Methods

### Search strategy

A literature search was carried out using Medline (1989 to June 2014), EMBASE (1947 to June 2014), Cochrane Library (1993 to June 2014), the China National Knowledge Infrastructure database (1979 to June 2014), the Chinese Biomedical Literature database (1990 to June 2014) and the Wanfang database (1982 to June 2014). The search terms used were (traditional Chinese medicine OR herbal medicine OR herbs OR herbal formula OR Chinese medicinal herb OR Shugan OR Jianpi OR Zhixie) AND (irritable bowel syndrome OR IBS) AND (randomized controlled trial AND (double-blind trial OR placebo-controlled trial)). No limit was placed on language. Manual searches of relevant studies supplemented the electronic searches.

### Study selection

Studies meeting the following criteria were included. (i) Patients were diagnosed with IBS-D. (ii) The study was performed as a randomized, double-blind, placebo-controlled, parallel-group trial that compared the efficacy of SJZT vs. placebo. (iii) Outcomes included at least one of the following: global symptom improvement, IBS Symptom Severity Scale (BSS) score and abdominal pain improvement. Global symptom improvement was recorded as primary outcome, and overall BSS score and abdominal pain improvement were recorded as secondary outcome measures.

### Data abstraction

Two researchers independently extracted data, including study design, randomization, diagnostic criteria for IBS-D, TCM criteria, sample size, dose and ingredients of each formula in the included studies, treatment duration, primary and secondary outcomes. Data were extracted as intention-to-treat analyses, in which drop-outs were assumed to be treatment failures, wherever trial reporting allowed this. Disagreements were resolved after discussion with other investigators. Assessment of methodological quality was conducted according to the Cochrane Collaboration tool.

### Data synthesis and analysis

Summary relative risk (RR) and 95% confidence intervals (CI) were reported for both global symptom improvement and abdominal pain improvement. Standardized mean difference (SMD) and 95% CI were reported for BSS scores. The χ^2^ test and the inconsistency index statistic (*I* ^2^) for heterogeneity were conducted [22]. If substantial heterogeneity occurred (*I* ^2^ >50% or *P*<0.05), a random effect model was used to calculate the pooled RR [23]. If there was no observed heterogeneity, the pooled RR was computed by using a fixed effect model. A sensitivity analysis was done to investigate potential sources of heterogeneity between studies through omitting one trial in turn. The number needed to treat (NNT) was calculated as the reciprocal of the therapeutic gain. Begg’s test was performed to evaluate publication bias [24]. Review Manager 5.1 and Stata 12.0 were used for analyses.

We calculated the optimal information size (OIS) to provide appropriate sample size for the meta-analysis [25]. To determine the OIS, a 60% control event rate for the chance of not improving symptomatic was presumed for the outcome global symptom improvement and a 25% RR reduction with a power of 80% and a two-sided α value of 0.01[26].

## Results

**Fig 1:**
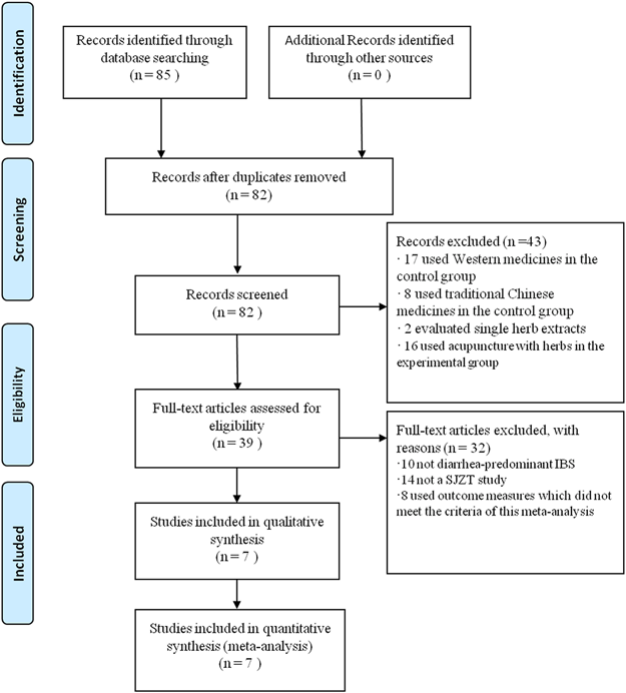
Flow chart of study selection process.

A total of 85 relevant studies were identified by computer search. Of these, three articles were duplicates and 43 articles were excluded on review of abstracts. After further reviewing, seven studies (*N* = 954) satisfied the inclusion criteria for the meta-analysis (Fig 1). A description of the included trial characteristics can be found in Table 1. The ingredients of herbal formulae were listed in Table 2.

**Table 1:** Characteristics of included studies.

Author	Criteria	Study Population	TCM criteria	Primary outcome	Secondary outcomes	*N* (SJZT vs. placebo)	SJZT	Dose	Duration
Bensoussan et al. 1998 (15)	Rome I	Two centers	N.R	1. BSS score 2. Global symptom improvement	Interference with life	43 vs. 35	Standard formula	Five capsules, t.i.d.	16 weeks
Leung et al. 2006 (12)	Rome II	Single center	stagnation of liver energy and deficiency of spleen	Global symptom improvement at 8 weeks	1. Global improvement at 4 weeks, 16 weeks 2. Individual symptom score 3. Daily bowel frequency 4. Bristol stool scales 5. Scales of the SF-36	60 vs. 59	Traditional Chinese herbal formula	One package, b.i.d.	8 weeks
Li et al. 2010 (16)	RomeIII	Single center	stagnation of liver energy and deficiency of spleen	1. BSS score 2. Global symptom improvement	1. Daily bowel frequency 2. Abdominal pain improvement	30 vs. 30	Chang Ji Tai granule	One package, b.i.d.	4 weeks
Chen et al. 2010 (17)	RomeIII	Multi-center	stagnation of liver energy and deficiency of spleen	Global symptom improvement	Abdominal pain improvement	329 vs. 113	Tong Xie Ning granule	5 g, t.i.d.	3 weeks
Tang et al. 2011 (18)	RomeIII	Single center	N.R	1. BSS score 2. Global IBS symptom improvement	IBS health-related QoL score	28 vs. 30	Chang An Yi Hao decoction	150 ml, t.i.d.	8 weeks
Cai et al. 2013 (19)	RomeIII	Single center	stagnation of liver energy and deficiency of spleen	BSS score	Syndrome of TCM score	18 vs. 19	Shu Gan Jian Pi Wen Shen decoction	150 ml, t.i.d.	8 weeks
Li et al. 2014 (20)	RomeIII	Single center	N.R	Global symptom improvement	1. Symptom improvement of mild IBS-D 2. Symptom improvement of moderate IBS-D 3. Symptom improvement of serious IBS-D	80 vs. 80	Chang Ji Tai granule	One package, b.i.d.	4 weeks

TCM, traditional Chinese medicine; BSS, symptom severity scale; QOL, quality of life questionnaire; N.R., not reported. The total number of patients included in this meta-analysis was 954 (SJZT vs placebo was 588 vs 366).

**Table 2:** The ingredients of each formula.

SJZT	Ingredients of each formula				
Bensoussan’s formula	*Codonopsis pilosulab* (Dang shen)	*Herba agastaches* (Huo xiang)	*Saposhnikovia divaricata* (Fang feng)	*Semen coicis* (Yi yi ren)	*Bupleuri chinensis* (Chai hu)
	*Herba artemisiae capillaris* (Yin chen)	*Magnoliae officinalis* (Hou pu)	*Atractylodes macrocephala* (Bai zhu)	*Citri reticulatae* (Chen pi)	*Zingiberis preparata* (Pao jiang)
	*Citri reticulatae immaturus* (Qin pi)	*Angelica dahurica* (Bai zhi)	*Phellodendri amurensis* (Huang bai)	*Poria cocos* (Fu ling)	*Plantaginis* (Che qian zi)
	*Paeoniae alba* (Bai shao)	*Aucklandiae* (Mu xiang)	*Glycyrrhizae preparata* (Zhi gan cao)	*Coptidis* (Huang lian)	*Schisandrae* (Wu wei zi)
Leung’s formula	*Astragalus membranaceus* (Huang qi)	*Paeoniae alba* (Bai shao)	*Atractylodes macrocephala* (Bai zhu)	*Bupleuri chinensis* (Chai hu)	*Atractylodes chinensis* (Cang zhu)
	*Citri reticulatae* (Chen pi)	*Murraya paniculata* (Jiu li xiang)	*Saposhnikovia divaricata* (Fang feng)	*Punica grantum* (Shi liu pi)	*Portulaca oleracea* (Ma chi xian)
	*Coptidis* (Huang lian)				
Li’s formula	*Paeoniae alba* (Bai shao)	*Glycyrrhizae preparata* (Zhi gan cao)	*Atractylodes macrocephala* (Bai zhu)	*Citri reticulatae* (Chen pi)	*Saposhnikovia divaricata* (Fang feng)
	*Fructus mume* (Wu mei)				
Chen’s formula	*Paeoniae alba* (Bai shao)	*Citri reticulatae immaturus* (Qin pi)	*Atractylodes macrocephala* (Bai zhu)	*Allii macrostemonis* (Xie bai)	
Tang ‘s formula	*Astragalus membranaceus* (Huang qi)	*Paeoniae alba* (Bai shao)	*Atractylodes macrocephala* (Bai zhu)	*Citri reticulatae* (Chen pi)	*Coptidis* (Huang lian)
	*Zingiberis preparata* (Pao jiang)	*Aucklandiae* (Mu xiang)	*Saposhnikovia divaricata* (Fang feng)	*Myristicae* (Rou dou kou)	
Cai’s formula	*Codonopsis pilosulab* (Dang shen)	*Paeoniae alba* (Bai shao)	*Atractylodes macrocephala* (Bai zhu)	*Poria cocos* (Fu ling)	*Curcumae wenyujin* (Yu jin)
	*Glycyrrhizae preparata* (Zhi gan cao)	*Alpiniae katsumadai* (Cao dou kou)	*Saposhnikovia divaricata* (Fang feng)	*Lablab album* (Bai bian dou)	*Citri reticulatae* (Chen pi)
	*Amomum villosum* (Sha ren)	*Albiziae* (He huan pi)	*Platycodon grandiflorum* (Jie geng)	*Semen coicis* (Yi yi ren)	

**Fig 2:**
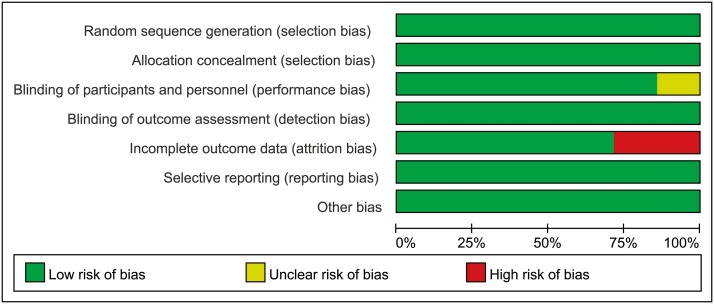
Risk of bias assessment.

The risk of bias assessment in the trials was summarized in Fig 2. A low risk of bias was found across studies for adequate sequence generation, allocation concealment, blinding of outcome assessment and selective outcome reporting. There were no details about placebo content in Tang et al. study [18]. Bensoussan et al.[15] and Cai et al.[19] studies were rated as high risk for the “incomplete outcome data” item ascribing to lack of intention-to-treat (ITT) analysis.

### Primary outcome: global symptom improvement

**Fig 3:**
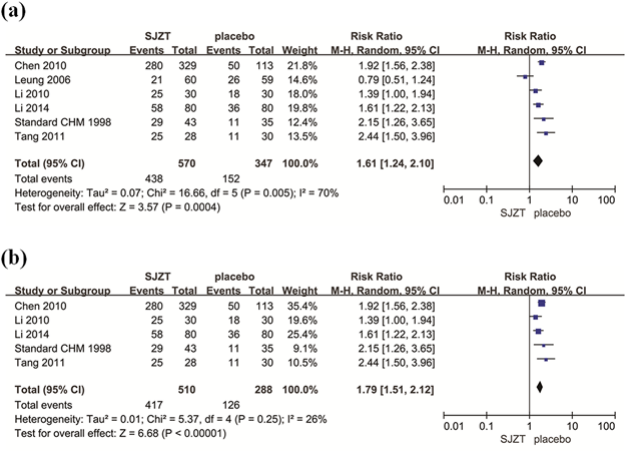
(a) Forest plot of primary outcomes, global symptom improvement with weights from random effects analysis. (b) Sensitivity analysis was performed by omitting one study.

**Fig 4:**
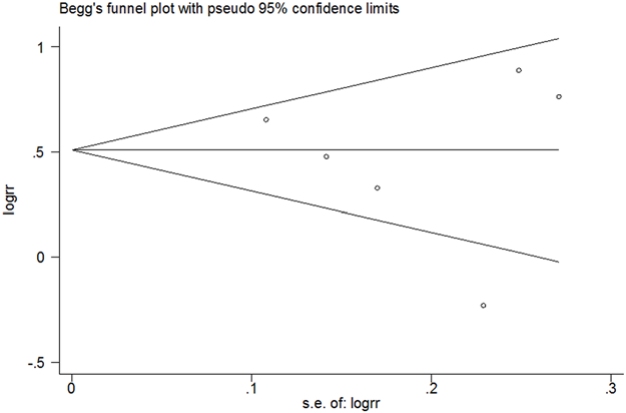
Funnel plot analysis of global symptom improvement (Begg’s test, *P* = 0.707). RR, relative risk.

Among the included studies, six evaluated global symptom improvement [12,15,16–18,20]: of 917 patients, 570 were assigned to the treatment groups, whereas 347 were assigned to the placebo groups. The number of participants included in this meta-analysis was slightly larger than the calculated OIS (917 vs. 794 patients). SJZT showed significant improvement in global symptoms compared to placebo (RR 1.61; 95% CI 1.24, 2.10; *P* = 0.0004) (Fig 3a). 76.8% of SJZT patients had global improvement over 43.8% of placebo patients (therapeutic gain = 33.0% with NNT = 3.0) (Table 3). The heterogeneity was significant (*P* = 0.005, *I* ^*2*^ = 70.0%). We performed a sensitivity analysis to investigate potential sources of heterogeneity and found that the Leung et al. study may be the main origin of heterogeneity in the meta-analysis [12]. The heterogeneity was small after exclusion of the Leung et al. study (*P* = 0.25, *I* ^*2*^ = 26%). However, the corresponding pooled RR was not conspicuously altered without the Leung et al. study (RR 1.79 95% CI 1.51, 2.12) (Fig 3b). The findings supported the robustness of the analysis. No evidence of asymmetry was identified by funnel plot analysis (Begg’s test *P* = 0.707) (Fig 4).

**Table 3:** Global symptom improvement, SJZT vs. placebo.

Study	Response rate, % (response/*N*)		Therapeutic gain, %	NNT	RR (95% CI)
	SJZT	Placebo			
Bensoussan et al,1998	67.4 (29/43)	31.4 (11/35)	36.0	2.8	2.15 (1.26, 3.65)
Leung et al,2006	35.0 (21/60)	44.1 (26/59)	-9.1	__	0.79 (0.51, 1.24)
Li et al,2010	83.3 (25/30)	60.0 (18/30)	23.3	4.3	1.39 (1.00, 1.94)
Chen et al,2010	85.1 (280/329)	44.2 (50/113)	40.9	2.4	1.92 (1.56, 2.38)
Tang et al,2011	89.3 (25/28)	36.7 (11/30)	52.6	1.9	2.44 (1.50, 3.96)
Li et al,2014	72.5 (58/80)	45.0 (36/80)	27.5	3.6	1.61 (1.22,2.13)
Pooled OR	76.8 (438/570)	43.8 (152/347)	33.0	3.0	1.61 (1.24, 2.10)

IBS, irritable bowel syndrome; NNT, number needed to treat; RR, relative risk.

### Secondary outcomes

#### Overall BSS score

**Fig 5:**
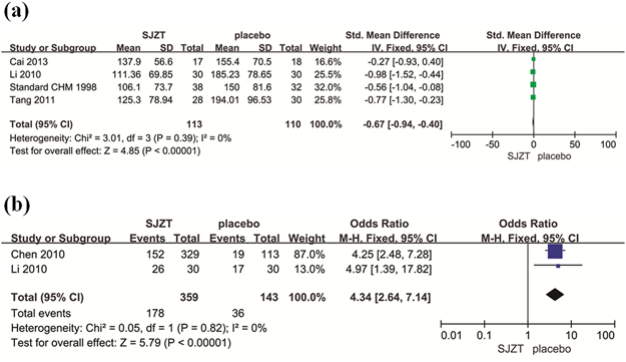
(a) Forest plot of secondary outcomes, overall BSS score with weights from fixed effects analysis. (b) Forest plot of secondary outcomes, abdominal pain improvement with weights from fixed effects analysis.

**Fig 6:**
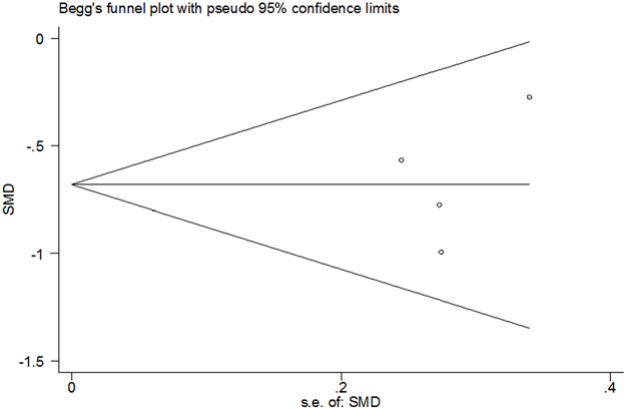
Funnel plot analysis of overall BSS score (Begg’s test, *P* = 1.000). RR, relative risk.

Four of seven studies used BSS to assess the severity of IBS symptoms [15,16,18,19]. SJZT reduced the overall BSS score compared with placebo (SMD—0.67; 95% CI—0.94, –0.40; *P*<0.00001) (Fig 5a). No substantial heterogeneity was found (*P* = 0.39, *I* ^2^ = 0.0%). Funnel plot analysis demonstrated no evidence of publication bias (Begg’s test, *P* = 1.000) (Fig 6).

#### Abdominal pain improvement

Two of the seven studies reported the outcome of abdominal pain improvement [16,17]. SJZT showed significant improvement for abdominal pain compared to placebo (RR 4.34; 95% CI 2.64, 7.14; *P*<0.00001) (Fig 5b). No observed heterogeneity existed (*P* = 0.82, *I* ^2^ = 0.0%).

Two included studies reported daily bowel frequency [12,16], but the data could not be incorporated because Li et al.[16] reported daily bowel frequency with means and SD values, while Leung et al.[12] reported the outcome as medians.

Two trials assessed health-related quality of life—one used the validated Hong Kong Chinese version of the short form 36 (SF-36) [12], while the other used the irritable bowel syndrome quality of life questionnaire (IBS-QOL) [18]. Both studies reported no significant difference in health-related quality of life between the SJZT group and the placebo group. Because of the heterogeneity of instruments, meta-analysis of the quality of life score could not be conducted.

### Safety profile and adverse events

Safety profile was evaluated in all the included studies. Four of these reported no adverse effects during SJZT treatment [16,18–20]. Bensoussan et al. reported that two patients in the standard formula group withdrew from the study associated with gastrointestinal discomfort [15]. In Leung et al.’s study, two participants suffered from skin rash and thyroiditis respectively in the treatment group and one participant suffered from facial nerve palsy in the placebo group [12]. Chen et al. reported two cases each of mild nausea and mild pruritus [17]. The adverse events of SJZT were no different from those of placebo.

## Discussion

The results from this meta-analysis revealed that SJZT showed significant improvement in global IBS symptoms (RR = 1.61), BSS score (SMD = –0.67), and abdominal pain (RR = 4.34) compared with placebo. No evidence of publication bias was found. Furthermore, the number of patients reporting adverse events of SJZT was similar to that of placebo.

A high placebo response rate exists in IBS patients. A series of meta-analyses showed that the proportion of placebo participants reporting global improvement was almost 40% [27–29], similar to the response effect of placebo in this meta-analysis (43.8%). The clinical benefit of SJZT for global IBS symptoms was significant, with therapeutic gains over placebo of 33.0% and NNT = 3.0. The reported NNT offered by 5-HT3 receptor antagonist was eight [30]. That being said, SJZT seems to be superior to 5-HT3 receptor antagonist for IBS-D. However, randomized, double-blind trials evaluating SJZT compared with 5-HT3 receptor antagonist for IBS-D have not been found in our search.

The pathogenesis of IBS has not been fully clarified. Numerous mechanisms involve in the development of IBS, including inflammation, gut mucosal immunology, disturbed gastrointestinal motility, gut microbes, visceral hypersensitivity, altered serotonin metabolism, and psychosocial distress [31–36]. Evidence for the effectiveness of SJZT for IBS-D was identified in modern pharmacological studies. Experimental data have demonstrated that *Chang ji tai* granule can relieve diarrhea symptoms, ameliorate the stress state, inhibit bowel motility, and reduce visceral hypersensitivity in rats with IBS-D, possibly by modulating the expression of substance P mRNA in the hypothalamus and colon [37,38]. *Chang an yi hao* decoction can significantly reduce visceral hypersensitivity and enhance anti-inflammatory activities by decreasing the expression of 5-HT and regulating the levels of anti-inflammatory cytokines in rats with IBS-D [39]. Further studies in vitro and in vivo of herbal formulae need to be conducted to better understand the drug mechanism

There was significant heterogeneity for the primary outcome. We performed a sensitivity analysis and found that the Leung et al. study may be the main origin of heterogeneity [12]. After exclusion of the Leung et al. study, the heterogeneity was effectively decreased while the corresponding pooled RR was not substantially altered. We checked all of the included studies carefully and found that there was difference of selection criteria of patients between Leung et al. study and the other selected studies. In Leung et al. study, diagnosis of IBS was based on Rome II criteria and the diarrhea-predominant type was defined by the author if diarrhea was present for at least 75% of the time during which a patient’s IBS was active [12]. The rigorous inclusion criterion of patients in Leung et al. study may contribute to the heterogeneity. Furthermore, we explored the pattern of herbal formula in Leung et al. study and found that Leung et al. added several herbs with heat-clearing functions based on SJZT, which maybe another important source of heterogeneity. Treatment based on syndrome differentiation is a characteristic of TCM. Of the included seven studies, the information for TCM syndrome classification was taken into consideration only in four studies [12,16,17,19]. For this reason, syndrome classification could be a matter of heterogeneity among the evaluated trials.

The methodological quality of included studies was general high. However, we did identified potential bias in two domains. Details about the chemical properties of placebo were not reported in Tang et al. study [18]. The extremely high response (89.3%) in the SJZT group in Tang et al. study may be related to spontaneous unblinding that the patient is aware of which treatment he or she is taking. The other flaw in the quality of included studies was the lack of ITT analysis, which could lead to incomplete outcome data. A total of 8 patients withdrew from the Bensoussan et al. study [15], of whom three patients were related to ineffective intervention. Cai et al. excluded two patients because of ineffective intervention [19]. Neither of these studies used ITT analysis and both of the studies were graded as high risk in incomplete outcome data. However, we extracted data as ITT analyses, assuming all drop-outs to be treatment failures, which could reduce the risk of attrition bias.

This meta-analysis had several limitations. First, the absolute number of clinical trials and the sample size of included studies were small. It needed to be demonstrated whether the effect size of SJZT would remain the same when applied in future large-scale trials. In the assessment of publication bias, the power of this meta- analysis was modest due to the small number of trials. Thus, there might be the possible existence of publication bias in our analysis. Second, in the included studies, patients in SJZT group were treated for 3 to 16 weeks under controlled conditions. The treatment duration was not long enough to evaluate the long term safety of SJZT for IBS-D. Third, only three of the included seven trials described follow-up evaluation [12,15,16]. Bensoussan et al. reported that, on follow-up 14 weeks after intervention completed, only the individualized formula treatment group maintained improvement rather than the standard formula group [15]. Because of the recurrent and chronic nature of IBS, long-term follow-up trials might yield different results. Therefore, it is necessary to specify the duration of the follow-up period with SJZT treatment. Fourth, discrepancy in formula composition, methods of preparation, and dose was observed between the studies, which may result heterogeneous.

In summary, the meta-analysis suggests that SJZT is more efficacious than placebo in improving global symptoms, BSS score, and abdominal pain for patients with IBS-D. SJZT is safe in short-term trials. However, due to the high clinical heterogeneity and small sample size of the included trials, further standardized preparation, randomized double-blind, multicenter, large-scale trials are required.

## Supporting Information

S1 PRISMA Checklist (DOC)
